# Evaluation of Genetic Polymorphisms in Patients with Multiple Chemical Sensitivity

**DOI:** 10.1371/journal.pone.0073708

**Published:** 2013-08-13

**Authors:** Xiaoyi Cui, Xi Lu, Mizue Hiura, Masako Oda, Wataru Miyazaki, Takahiko Katoh

**Affiliations:** Department of Public Health, Faculty of Life Sciences, Kumamoto University, Kumamoto, Japan; Indian Institute of Toxicology Reserach, India

## Abstract

**Objective:**

Multiple chemical sensitivity (MCS) is a chronic medical condition characterized by symptoms that the affect an individual’s response to low-level chemical exposure. In this study, we identified a chemical sensitive population (CSP) and investigated the effect of genetic polymorphisms on their risk of chemical sensitivity.

**Methods:**

A quick environment exposure sensitivity (QEESI) questionnaire was used to survey 324 Japanese male workers whose DNA samples had been collected and stored. The following genes, which encode enzymes affecting the metabolic activation of a large number of xenobiotic compounds, were selected and analyzed in order to determine their influence on genetic predisposition to CSP: cytochrome P450 (CYP) 2E1, N-acetyl transferase (NAT) 2, glutathione S-transferase (GST) M1, GSTT1, GSTP1, low Km aldehyde dehydrogenase (ALDH2), and superoxide dismutase (SOD) 2.

**Results:**

Significant case-control distributed differences were observed in *SOD2* polymorphisms and allele frequency distribution in high chemical sensitive subjects. Both the significant adjusted OR of 4.30 (95% CI, 1.23–15.03) and 4.53 (95% CI, 1.52-13.51) were observed in *SOD2* Ala/Ala and Val/Ala compared to Val/Val and in *SOD2* Ala/Ala compared to Val/Ala compared to Val/Val genetic analysis in the high chemical sensitivity case-control study.

**Conclusions:**

We observed that high chemical sensitive individuals diagnosed by using Japanese criteria as MCS patients were more significantly associated with *SOD2* polymorphisms.

## Introduction

Individuals experiencing multiple chemical sensitivity (MCS) often report symptoms from various organs related to inhalation of multiple unrelated airborne chemicals in concentrations below what is normally associated with toxicological responses [1]. MCS is a chronic medical condition characterized by symptoms that affect an individual’s responses to low-level chemical exposure. These chemicals can include pesticides, plastics, and paint fumes. Symptoms are usually vague and nonspecific, such as fatigue and headaches. In general the reported symptoms are attributed to previous chemical exposure and recur on subsequent exposure to similar or structurally unrelated chemicals at levels normally considered to be nontoxic [2]. The clinical characteristics of MCS patients are usually evaluated using questionnaires such as the environmental exposure and sensitivity inventory (EESI) questionnaire or clinical interviews that rely on the subject’s retrospective self-reports [3]. Miller and Prihoda developed a globally standardized self-administered questionnaire, the quick environment exposure sensitivity (QEESI), which is designed to assist researchers and clinicians in screening, studying, and evaluating patients with MCS [4].

The variation in individual responses to multiple environmental chemicals is exceptionally wide. This variation is accounted for by differences in metabolic capacity, DNA repair capacity, or genetic predisposition. The existence of several well-known genetic polymorphisms affecting the activity of enzymes metabolizing xenobiotics have prompted research into whether these polymorphisms are associated with MCS and chemical sensitivity in general populations [5–8]. Either negative or significant association in genetic polymorphisms with MCS or self-reported chemical-related sensitivity was found in those studies.

Chemicals that enter cells are subjected to biotransformation by oxidative phase I enzymes in the cytoplasm as cytochrome P450 (CYP) s. CYP2E1 represents a major CYP isoform in the human liver and is also expressed in extrahepatic tissue. It can be induced by certain chemicals such as ethanol although large interindividual variations have been observed in its induction, suggesting that genetic polymorphisms may be involved [9]. A polymorphism in *Rsa*I, which is located in the 5′-flanking region of *CYP2E1* gene, was reported to be associated with the transcriptional regulation of gene expression [10]. The N-acetyl transferase (NAT) 2 enzyme plays an important role in the metabolism of aromatic and heterocyclic amines via *N*-acetylation and *O*-acetylation pathways, which are responsible for their activation and/or deactivation, respectively [11]. Functional variation of NAT2 leads to a slow or rapid acetylator phenotype. A study by Vineis et al. indicated that clearance of low-dose environmental carcinogens decreases the slow-acetylator phenotype of NATs [12]. Glutathione S-transferase (GST) s are multifunctional enzymes and the variant allele changes of these genes result in either total absence or a substantial change in enzyme activity [13]. The absence of GSTM1 and GSTT1 phenotypic activity is caused by homozygosity for an inherited deletion of these genes, referred to as the null genotype [14]. The Ile105Val (A to G substitution replacing isoleucine with valine polymorphism) in the *GSTP1* gene has been found to modify the enzyme’s specific activity and affinity for electrophilic substrates [15]. Low Km aldehyde dehydrogenase (ALDH2) predominantly located in mitochondria is characterized by being responsible for the oxidation of most of the acetaldehyde generated during alcohol metabolism [16]. Approximately half of the Japanese population lacks ALDH2 activity, and the enzyme deficit of ALDH2 causes a significant change in the rate of ethanol metabolism [17]. This is often caused by the ALDH ^*^2(ALDH2_K504_) mutant allele. Individuals possessing either 1 or 2 copies of the mutant allele show alcohol-related sensitivity responses, including facial flushing and hangovers [18].

Many lifestyles are closely associated with oxidative stress, which is augmented by smoking, drinking, and an irregular diet. Many environmental factors can also generate oxygen radicals that induce DNA damage and reactive oxygen species (ROS) production [19]. Superoxide dismutase (SOD) enzymes act as antioxidants and protect cellular components from being oxidized by reactive oxygen species (ROS) [20]. SOD plays a pivotal role in protecting cells from free radicals and oxidative damage. SOD2 is one of the major superoxide scavengers in mitochondria, where it catalyzes accumulated superoxide radicals into H_2_0_2_ [21]. The 47 cytosine-to-thymine (47C→T) transition at codon 16 in the *SOD2* gene creates a sense mutation of alanine-to-valinein in the SOD2 protein [22].

## Materials and Methods

### Study Characteristics

The present study was conducted at a paper pulp producing company between 2002 and 2006 in Kyushu, Japan. A total of 324 male subjects whose purified DNA had been obtained and stored by the company were asked to complete QEESI questionnaires and were eligible for this study. Subjects with a diagnostic history of cancer, chronic obstructive lung disease, cardiovascular disorder or diabetes were excluded from the study. All study subjects completed the QEESI questionnaire, which also covered the history of drinking and smoking. The distribution of smoking, drinking, age, diagnosis history, score status of survey subscales, and the CSP-diagnosed criterion of the participants are presented in Table 1.

**Table 1:** Characteristics of the entire study population and the distribution of QEESI subscales.

		**n**	**%**
**Age**	<40	83	25.6%
	40–49	95	29.3%
	≥50	146	45.1%
**Average age** (years ± SD^a^)	46.84 ± 8.79		
**Smoking**	Non-smoker	137	42.3%
	Smoker	187	57.7%
**Drinking**	Non-drinker	62	19.1%
	Drinker	262	80.9%
**History of diagnosis**	Multiple chemical sensitivity (MCS)	3	0.9%
	Allergy	61	18.8%
	Sick house	0	0.0%
	Non	260	80.3%
**QEESI** ^b^ **subscales**			
Chemical sensitivity	0 score	113	34.9%
	1–39 score	182	56.2%
	≥40 score (cut-off value)	29	8.9%
Symptom severity	0 score	53	16.4%
	1–19 score	182	56.1%
	≥20 score (cut-off value)	89	27.5%
Life impact	0 score	176	54.3%
	1–9 score	90	27.8%
	≥10 score (cut-off value)	58	17.9%
**CSP** ^c^ **cases**	All	116	100%
Low chemical sensitivity	Cut-off value subscale = 1	67	57.8%
Middle chemical sensitivity	Cut-off value subscale = 2	38	32.7%
High chemical sensitivity	Cut-off value subscale = 3	11	9.5%
**Controls**	Cut-off value subscale = 0	208	64.2%

^a^. Standard deviation

^b^. Quick Environment Exposure Sensitivity

^c^. Chemical sensitive population

### Survey instruments

We used the QEESI questionnaire (Japanese version) translated by Ishikawa and Miyata for the survey [23]. We used this diagnostic instrument to define the chemical sensitive population (CSP) in this study and divided them into 3 case groups according to the scores achieved by cut-off values of the QEESI subscales. Each criterion subscale of QEESI has 10 questions, and each question has a possible score of 0–10. Therefore, the total possible score of each subscale was 0–100.

The chemical sensitivity section of the survey asked the subjects to list various odors or chemical exposures that made them feel sick (items like diesel or gas engine exhaust; gasoline; or insecticide). The symptom severity section asked about symptoms the subjects may have experienced commonly (items like problems with muscles or joints, such as pain, aching, cramping, stiffness, or weakness; problems with your head, such as headaches or a feeling of pressure or fullness in your face or head; problems with your skin, such as a rash, hives, or dry skin). The life impact section of the survey asked if the subjects were sensitive to certain chemicals or foods and if the sensitivities had affected various aspects of their life (items like your diet; your ability to work or go to school; your choice of clothing).

Hojo et al. designed a study to establish the cut-off value for Japanese using the QEESI for screening MCS patients [24]. The cut-off values for Japanese subjects were determined to be ≥40 for the chemical sensitivity subscale, ≥20 for the symptom severity subscale, and ≥10 for the life impact subscale. By using these 3 criteria, we divided our subjects into 3 case groups and 1 control group. Individuals who were 1 subscale from the cut-off value were defined as low chemical sensitivity; those who were 2 subscales from the cut-off value were defined as middle chemical sensitivity; those who were 3 subscales from the cut-off value were defined as high chemical sensitivity; those who were 0 subscales from the cut-off value were defined as the controls. Scores for each subscale in the controls were as follows: chemical sensitivity <40, symptom severity <20 and life impact <10. Therefore, they were not compliant with any of the diagnostic criteria in QEESI, and were not diagnosed with MCS before enrolment in this study. In addition to QEESI, the diagnosis history of MCS, sick house syndrome, allergic disease, and age were also included in the study survey.

### Genotyping

#### DNA isolation

Genomic DNA was isolated from whole blood using the Viogene^®^ Blood and Tissue Genomic DNA Extraction Miniprep system (Viogene, Japan) according to the manufacturer’s instructions and stored at −20° C.

#### Genotyping assay

Genes encoding enzymes affecting the biotransformation of a large number of xenobiotic compounds were selected for determination of their ability to predispose to CSP, namely, CYP2E1, NAT2, GSTM1, GSTT1, GSTP1, ALDH2, and SOD2.

The *CYP2E1* genetic polymorphism was determined by polymerase chain reaction (PCR) amplification run on a GeneAmp PCR System 9700 (Applied Biosystems), followed by digestion with *Rsa*I (Takara Bio, Japan) since the predominant allele (C1) is sensitive while the C2 allele is resistant to *Rsa*I digestion [9]. The *CTP2E1* genotypes were categorized as the homozygous genotype of C1/C1 or C2/C2 and the heterozygous genotypes of C1/C2. The *NAT2* alleles examined were WT (^*^4, wild-type), M1 (^*^5), M2 (^*^6), and M3 (^*^7). The ^*^5, ^*^6, and ^*^7 single nucleotide variations have all been associated with decreased enzyme activity [25]. The NAT2 single nucleotide polymorphisms (SNPs) of ^*^6 (rs1799930, Applied Biosystems assay ID:C__1204091_10) and ^*^7 (rs1799931, Applied Biosystems assay ID:C__572770_20) alleles were analyzed by real-time PCR. The ^*^5 allele was analyzed by PCR restriction fragment length polymorphism (PCR-RFLP) and digestion with the restriction enzyme *Kpn*I (Takara Bio, Japan). The NAT2 rapid acetylator genotypes are those with none mutant alleles (^*^4/ ^*^4); slow acetylator genotypes are those with 2 mutant alleles (^*^5/ ^*^5, ^*^5/ ^*^6, ^*^5/ ^*^7, ^*^6/ ^*^6, ^*^6/ ^*^7, and ^*^7/ ^*^7); intermediate acetylator genotypes are those with 1 mutant allele (^*^4/ ^*^5, ^*^4/ ^*^6, and ^*^4/ ^*^7). A multiplex PCR method was used to detect the presence or absence of GSTM1 and GSTT1. In this method, both the GST and β-globin primers (Sigma-Aldrich, Japan) are included in the same PCR reaction. The absence of a 219-bp band indicates the *GSTM1* null genotype, and the absence of a 480-bp band indicates the *GSTT1* null genotype; β-globin was coamplified in all samples [14]. The *GSTP1* genotype was determined by using PCR-RFLP as previously described [26]. The *GSTP1* genotypes were categorized as the homozygous genotypes of A/A or G/G and the heterozygous genotype of A/G. The *ALDH2* E504K polymorphism (SNP: rs671, Applied Biosystems assay ID:C__11703892_10), and the *SOD2* Val16Ala polymorphism (SNP: rs4880, Applied Biosystems assay ID:C__8709053_10) were analyzed by real-time PCR. All real-time PCR reactions were performed in 48-well plates on a StepOne Real-Time PCR system (Applied Biosystems) and were prepared by using a TaqMan^®^ Universal PCR Master Mix, TaqMan^®^ SNP genotyping assay mix, DNase-free water, and 10 ng of genomic DNA in a final volume of 10 μL per well. The cycling conditions were 30 s at 60° C, 10 min at 95° C, 35 cycles of 15 s at 95° C and 1 min at 60° C, with a final step of 30 s at 60° C. The standard mode reaction time was 90 min. The allelic discrimination results were determined after the amplification by performing an end-point read in an allelic discrimination graph (VIC on abscissa, FAM on ordinate). The genotypes of *ALDH2* were categorized as the homozygous genotypes of ^*^1/ ^*^1 or ^*^2/ ^*^2 and the heterozygous genotype of ^*^1/ ^*^2. The genotypes of *SOD2* were categorized as the homozygous genotypes of Val/Val or Ala/Ala and the heterozygous genotype of Val/Ala.

### Statistical analysis

Statistical power calculations were performed using Epistat (Finnish Institute of Occupational Health). The study sample size had at least 80% power (two-sided test signiﬁcant, α of 0.05) to detect an OR of at least 2.5, following the calculations used in previous studies [9,14,16,25–27]. The distribution of each genetic variant in the cases and controls was first assessed for consistency with the Hardy-Weinberg equilibrium (HWE) on a contingency table of observed-versus-predicted genotype frequencies by using Chi-Square Goodness of Fit test.

The frequency distribution of genotypes and alleles in cases and controls was determined by analysis using the Pearson’s chi-square test or Fisher’s exact test. To determine an association between each SNP and CSPs, we computed the overall genotypic test of association in the dominant or recessive genetic models and in the additive genetic models. Relative association in the case-control designs were assessed by calculating odds ratios (ORs) and 95% confidence intervals (95% CIs) in logistic regression analyses; two-tailed *P* values <0.05 were considered statistically significant. Statistical analysis was carried out using SPSS version 18 (SPSS, Japan).

### Ethical Statement

The Ethics Review Boards of Miyazaki University (no. 82; April 9, 2003) and Kumamoto University (no. 168; May 11, 2011) approved this study, following the ethical guidelines for human genome research. All participants provided their written informed consent to participate in this study, and the complete protection of their personal data was agreed in a written form.

## Results

Table 1 presents the diagnostic history frequencies of MCS, sick house syndrome, and allergic diseases of the study participants. Three subjects reported a history of diagnosis for MCS. None of the subjects in this study population had been diagnosed with sick house syndrome. The percentage of subjects that had QEESI cut-off values applying to the subscales of chemical sensitivity, symptom severity, and life impact were 8.9%, 27.5%, and 17.9% respectively. In CSP cases, 57.8% of the subjects were defined as low chemical sensitivity; 32.7%, as middle chemical sensitivity; and 9.5%, as high chemical sensitivity. Individuals previously diagnosed with MCS were all defined as the CSP case group, one of them was classified as high chemical sensitivity.

The genotype distributions of *CYP2E1*, *NAT2*, *GSTP1*, *ALDH2*, and *SOD2* in all the cases and controls were checked and found to not differ significantly from those predicted by the Hardy–Weinberg law (*p* > 0.05), indicating no evidence of non-random selection. The Hardy-Weinberg equilibrium could not be tested in *GSTM1* and *GSTT1* polymorphisms because of the inability of the present PCR protocol to separate heterozygous carriers of the deletion polymorphisms. There was no significant difference in the distribution of drinking and smoking statuses between cases and controls (Table 2). The proportion of low chemical sensitive individuals (Case 1) who were over 50 years old was significantly greater in the cases than in the controls (56.7% vs. 39.9%). There was no significant difference in the mean age between Case 2 group and control group, also no significant difference was found between Case 3 group and control group (*p* > 0.05, student’s t test, data not shown). Significant frequency differences in *SOD2* genotypes and alleles were observed in high chemical sensitive individuals compared to controls. Instead of the genotype, only the allele-frequency difference in *GSTP1* was observed in middle chemical sensitive individuals compared to controls.

**Table 2:** Distribution of age, smoking, drinking, and genotypes in chemical sensitive population (CSP) cases and controls.

		**Controls (%)**	**Case 1 (%)**	**P^a,b^**	**Case 2 (%)**	**P^a,b^**	**Case 3 (%)**	**P^a,c^**
			Low chemical sensitivity		Middle chemical sensitivity		High chemical sensitivity	
		n = 208	n = 67		n = 38		n = 11	
**Age**	<40	61 (29.3%)	9 (13.4%)		9 (23.7%)		4 (36.4%)	
	40–49	64 (30.8%)	20 (29.9%)		9 (23.7%)		2 (18.2%)	
	≥50	83 (39.9%)	38 (56.7%)	0.02	20 (52.6%)	0.34	5 (45.4%)	0.74
**Smoking**	Non-smoker	83 (39.9%)	31 (46.3%)		16 (42.1%)		7 (63.6%)	
	Smoker	125 (60.1%)	36 (53.7%)	0.36	22 (57.9%)	0.80	4 (36.4%)	0.11
**Drinking**	Non-drinker	38 (18.3%)	12 (17.9%)		10 (26.3%)		2 (18.2%)	
	Drinker	170 (81.7%)	55 (82.1%)	0.95	28 (73.7%)	0.25	9 (81.8%)	0.68
**NAT2**								
Genotype	Rapid	90 (43.3%)	36 (53.7%)		20 (52.6%)		7 (63.6%)	
	Inter + Slow	118 (56.7%)	31 (46.3%)	0.14	18 (47.4%)	0.29	4 (36.4%)	0.16
**GSTM1**								
Genotype	non-null	121 (58.2%)	37 (55.2%)		18 (47.4%)		4 (36.4%)	
	homozygous-null	87 (41.8%)	30 (44.8%)	0.67	20 (52.6%)	0.22	7 (63.6%)	0.13
**GSTT1**								
Genotype	non-null	84 (40.4%)	31 (46.3%)		19 (50.0%)		5 (45.5%)	
	homozygous-null	124 (59.6%)	36 (53.7%)	0.40	19 (50.0%)	0.27	6 (54.5%)	0.49
**GSTP1**								
Genotype	A/A	154 (74.0%)	48 (71.6%)		23 (60.5%)		6 (54.5%)	
	G/G + A/G	54 (26.0%)	19 (28.4%)	0.70	15 (39.5%)	0.09	5 (45.5%)	0.14
Allele	A	358 (86.1%)	114 (85.1%)		58 (76.3%)		17 (77.3%)	
	G	58 (13.9%)	20 (14.9%)	0.78	18 (23.7%)	0.03	5 (22.7%)	0.20
**CYP2E1**								
Genotype	C1/C1	117 (56.2%)	39 (58.2%)		27 (71.1%)		8 (72.7%)	
	C2/C2 + C1/C2	91 (43.8%)	28 (41.8%)	0.78	11 (28.9%)	0.09	3 (27.3%)	0.23
Allele	C1	307 (73.8%)	102 (76.1%)		62 (81.6%)		19 (86.4%)	
	C2	109 (26.2%)	32 (23.9%)	0.59	14 (18.4%)	0.15	3 (13.6%)	0.19
**ALDH2**								
Genotype	^*^1/^*^1	125 (60.1%)	39 (58.2%)		25 (65.8%)		9 (81.8%)	
	^*^2/^*^2 + ^*^1/^*^2	83 (39.9%)	28 (41.8%)	0.78	13 (34.2%)	0.51	2 (18.2%)	0.13
Allele	^*^1	326 (78.4%)	105 (78.4%)		60 (78.9%)		20 (90.9%)	
	^*^2	90 (21.6%)	29 (21.6%)	1.00	16 (21.1%)	0.91	2 (9.1%)	0.12
**SOD2**								
Genotype	Val/Val	159 (76.4%)	52 (77.6%)		28 (73.7%)		5 (45.5%)	
	Ala/Ala + Val/Ala	49 (23.6%)	15 (22.4%)	0.84	10 (26.3%)	0.71	6 (54.5%)	0.03
Allele	Val	365 (87.7%)	116 (86.6%)		65 (85.5%)		15 (68.2%)	
	Ala	51 (12.3%)	18 (13.4%)	0.72	11 (14.5%)	0.59	7 (31.8%)	0.02

^a^. *p* value <0.05 is considered statistically significant

^b^. Pearson′s chi-square test

^c^. Fisher’s exact test

In addition, we determined the association between genetic polymorphisms and CSP risk in the 3 case-control designs using logistic regression analyses as shown in Table 3. Both crude ORs and adjusted ORs (adjusted by age, smoking, and drinking) were calculated. In the high chemical sensitivity (Case 3) case-control study, the significant crude OR of 3.90 (95% CI, 1.14–13.31, *p* = 0.03) and the adjusted OR of 4.30 (95% CI, 1.23–15.03, *p* = 0.02) was observed in *SOD2* Ala/Ala and Val/Ala variants compared to the Val/Val genotype. The significant crude OR of 3.67 (95% CI, 1.32-10.20, *p* = 0.01) and adjusted OR of 4.53 (95% CI, 1.52-13.51, *p* = 0.01) were observed in *SOD2* additive genetic model analysis of Ala/Ala vs. Val/Ala vs. Val/Val in the Case 3 study. The *GSTP1* genetic analysis of G/G vs. A/G vs. A/A obtained a significant crude OR in the Case 2 study. However, the OR value decreased and lost statistical significance after adjusting by age, smoking, and drinking status.

**Table 3:** Odds ratios of chemical sensitive population (CSP) cases compared to controls categorized by genotype.

**Variable** ^a^	**Control**	**Case 1 (Low chemical sensitivity)**				**Case 2 (Middle chemical sensitivity)**				**Case 3 (High chemical sensitivity)**			
	n=208	n=67				n=38				n=11			
		OR (95% CI)	P^c^	OR^b^ (95% CI)	P^c^	OR (95% CI)	P^c^	OR^b^ (95% CI)	P^c^	OR (95% CI)	P^c^	OR^b^ (95% CI)	P^c^
		Crude		Adjusted		Crude		Adjusted		Crude		Adjusted	
**NAT2**													
Genotype Slow + Inter vs. Rapid	1^d^	0.66 (0.38–1.14)	0.14	0.68 (0.38–1.19)	0.17	0.69 (0.34–1.37)	0.29	0.68 (0.34–1.36)	0.27	0.44 (0.12–1.54)	0.20	0.48 (0.13–1.70)	0.25
Slow vs. Inter vs. Rapid	1^d^	0.70 (0.43–1.12)	0.13	0.70 (0.43–1.14)	0.15	0.68 (0.37–1.24)	0.21	0.66 (0.36–1.22)	0.18	0.43 (0.13–1.38)	0.16	0.47 (0.14–1.51)	0.20
**GSTM1**													
Genotype homozygous-null vs. non-null	1^d^	1.13 (0.65–1.96)	0.67	1.09 (0.62–1.93)	0.76	1.55 (0.77–3.09)	0.22	1.51 (0.75–3.04)	0.25	2.43 (0.69–8.57)	0.17	2.34 (0.66–8.30)	0.19
**GSTT1**													
Genotype homozygous-null vs. non-null	1^d^	0.79 (0.45–1.37)	0.40	0.75 (0.42–1.32)	0.32	0.68 (0.34–1.36)	0.27	0.66 (0.33–1.33)	0.24	0.81 (0.24–2.75)	0.81	0.84 (0.25–2.87)	0.78
**GSTP1**													
Genotype G/G + A/G vs. A/A	1^d^	1.13 (0.61–2.09)	0.70	1.06 (0.57–1.98)	0.86	1.86 (0.91–3.82)	0.09	1.76 (0.85–3.64)	0.13	2.38 (0.70–8.10)	0.17	2.48 (0.72–8.55)	0.15
G/G vs. A/G vs. A/A	1^d^	1.08 (0.62–1.89)	0.78	1.02 (0.58–1.81)	0.93	1.88 (1.04–3.41)	0.04	1.79 (0.98–3.28)	0.06	1.85 (0.64–5.31)	0.26	1.95 (0.66–5.75)	0.23
**CYP2E1**													
Genotype C2/C2 + C1/C2 vs. C1/C1	1^d^	0.92 (0.53–1.61)	0.78	0.96 (0.54–1.71)	0.90	0.52 (0.25–1.11)	0.09	0.50 (0.24–1.08)	0.08	0.48 (0.12–1.87)	0.29	0.52 (0.13–2.06)	0.35
C2/C2 vs. C1/C2 vs. C1/C1	1^d^	0.89 (0.58–1.38)	0.61	0.94 (0.59–1.47)	0.78	0.67 (0.37–1.20)	0.18	0.65 (0.35–1.18)	0.16	0.47 (0.14–1.57)	0.22	0.50 (0.15–1.67)	0.26
**ALDH2**													
Genotype ^*^2/^*^2 + ^*^1/^*^2 vs. ^*^1/^*^1	1^d^	1.08 (0.62–1.89)	0.78	1.02 (0.55–1.89)	0.95	0.78 (0.38–1.62)	0.51	0.61 (0.27–1.36)	0.22	0.34 (0.07–1.59)	0.17	0.26 (0.05–1.40)	0.12
^*^2/^*^2 vs. ^*^1/^*^2 vs. ^*^1/^*^1	1^d^	1.00 (0.61–1.65)	1.00	0.95 (0.54–1.67)	0.85	0.97 (0.53–1.77)	0.91	0.78 (0.40–1.53)	0.47	0.34 (0.08–1.53)	0.16	0.27 (0.05–1.35)	0.11
**SOD2**													
Genotype Ala/Ala + Val/Ala vs. Val/Val	1^d^	0.94 (0.49–1.81)	0.84	0.89 (0.46–1.75)	0.74	1.16 (0.53–2.55)	0.71	1.09 (0.49–2.42)	0.84	3.90 (1.14–13.31)	0.03	4.30 (1.23–15.03)	0.02
Ala/Ala vs. Val/Ala vs. Val/Val	1^d^	1.11 (0.63–1.96)	0.72	1.03 (0.58–1.85)	0.91	1.22 (0.60–2.50)	0.59	1.11 (0.54–2.30)	0.78	3.67 (1.32–10.20)	0.01	4.53 (1.52–13.51)	0.01

^a^. OR, odds ratio; 95% CI, 95% confidence interval

^b^. Odds Ratios were adjusted by age, smoking, and drinking

^c^. *p* value <0.05 is considered statistically significant

d. Reference category

## Discussion

This study focused on determining if there were any associations between chemical sensitivity and genetic polymorphisms. There were no significant associations between chemical sensitivity and the genetic polymorphisms in the previously studied genes—*NAT2, GSTM1*, *GSTT1*—although there was a significant association between middle chemical sensitivity and the genetic polymorphism in *GSTP1* before adjusting for other confounding factors. In addition, no significant results were obtained when the genetic polymorphisms in *CYP2E1* and *ALDH2* were compared to chemical sensitivity. However, results from this case-control study indicated that there was an increased risk of high chemical sensitivity associated with *SOD2* Ala allele genotypes. The diagnostic criterion for high chemical sensitivity fit with all the 3 cut-off values of the QEESI subscales that are used to diagnose MCS patients using Japanese criteria [24].

CYP2E1 is a major contributor to ethanol-induced oxidant stress and to ethanol-induced liver injury [28]. Individuals with the C2/C2 genotype have higher expression of CYP2E1 mRNA than C1/C1 genotype subjects [29]. Although we predicted that the genetic variant of CYP2E1 might be associated with ethanol-induced CSP, no significant association was found. This may be due to the fact that even though the participants in this study were slightly exposed to ethanol-related xenobiotic chemicals such as alcohol, it did not lead to excessive oxidant stress. The *NAT2* genetic polymorphisms associated with chemical sensitivity have been investigated in several studies with inconsistent results. The effect of NAT2 activity was not found in the case-control study of MCS by Berg et al [5]. However, the fast NAT2 acetylator was associated with self-reported chemical sensitivity only in the most severely affected group (OR = 3.1, *p* = 0.04) [5]. The study by McKeown-Eyssen et al. also showed that NAT2 rapid acetylator (OR = 4.14; *p* = 0.01) was significantly higher in MCS cases than in controls [8]. The *NAT2* slow or inter compared to rapid acetylator genotype showed gradually lower OR from low to high chemical sensitivity cases in this study, but no statistical significance was found. This may be due to the different characteristics of the research subjects; the genetic polymorphisms of *NAT2* may not have been sensitive enough in our case-control designs. *GST* genetic polymorphisms have been associated with atopy (allergy, asthma, and atopic dermatitis) [13]. A study by Mapp et al. showed that the *GSTP1* genetic polymorphism is associated with asthma and airway hyperresponsiveness [30]. Schnakenberg et al. suggested that *GSTM1* and *GSTT1* genes were significantly deleted homozygously more often (homozygous-null) in self-reported chemical sensitivity cases than in controls, although no case-control differences were observed in the genotype frequencies of *GSTP1* [7]. No significant differences between the genetic polymorphisms of *GSTT1* and *GSTM1* were observed in the present study, but a gradually higher OR was found in *GSTM1* homozygous-null compared to non-null genotype from low to high chemical sensitivity cases. In the case 2 study, significant results were observed in the *GSTP1* allele frequency and in the crude OR, although the logistic regression analysis lost significance after adjusting for confounding factors. In a review study, cleaning agents, pesticides, perfumes, and vehicle exhaust were the products most often reported to trigger MCS that were not due to smoking and drinking [31]. The triggers of symptoms of middle chemical sensitivity cases defined by this study may not be those from the most often reported MCS trigger but were also influenced by multiple factors such as age, smoking and drinking which may be predominantly associated with the *GSTP1* genetic polymorphism. Further research and larger sample sizes will be necessary to evaluate the statistical significance of the interaction between the *GSTP1* genetic polymorphisms and CSPs by stratification according to these multiple factors. Alcohol consumption is associated with many health problems, including alcohol-related metabolic syndrome and hypertension [32]. The SNP rs671 in the ALDH2 gene showed the strongest association with drinking behavior in Japanese samples [33]. We failed to find a significant association between *ALDH2* or drinking status and CSP risk. This may be due to the fact that alcohol consumption was very common in the research participants, which were composed of mostly middle-aged men.

Overexpression of SOD2 is associated with increased levels of H_2_O_2_, a major contributor to oxidative stress [34]. In addition, H_2_O_2_ can also be transformed into hypochlorous acid (HOCl) through a myeloperoxidase (MPO)-catalyzed reaction, thus inducing cell damage [35]. The Ala-variant of *SOD2* allows more efficient SOD2 importation into the mitochondria. This generates more active SOD2 compared with the Val-variant and is related to the induction of oxidative stress [36,37]. We predicted that the *SOD2* genetic polymorphismis related to oxidative stress and associated with the CSP risk. In the high chemical sensitivity group, a significantly high crude OR of 3.67 was observed in the *SOD2* Ala allele compared to the Val allele carriers in the additive genetic analysis, and increased to 4.53 after adjusting for other confounding factors. This result may indicate that the Ala-variant genotypes are associated with elevated SOD2 activity together with increasing oxidative stress and increased chemical sensitivity risk.

Furthermore, in a clinical-based investigation, women were found to be more susceptible to MCS than men [5,38]. In contrast, a population-based investigation found no gender differences for MCS [39]. The CSP research subjects in this study were composed entirely of men, unlike the study population in previous studies. We did not match age status to determine the age-distribution differences in the case control studies in reference to a previous study, which stated that MCS patients were found to be significantly older when compared to controls [5]. In our study, the proportion of individuals over 50 years old was greater in the low chemical sensitivity cases, but no significant age difference was found in the middle or high chemical sensitivity cases. In particular, we did not select the MCS patients diagnosed in hospital as cases in this study. We are currently investigating the chemical sensitivity status and the genetic polymorphisms related to chemical sensitivity in a normal working population.

## Conclusion

The aim of this study was to examine the association between chemical sensitivity and genetic polymorphisms in *CYP2E1, NAT2*, *GSTM1*, *GSTT1*, *GSTP1*, *ALDH2*, and *SOD2*. In conclusion, we observed that the high chemical sensitive individuals who were also diagnosed by Japanese QEESI criteria as MCS patients were more significantly associated with *SOD2* polymorphisms. We hypothesize that our results reflect the gene-environment associations of increased chemical sensitivity in individuals, but further studies are needed to verify our observations. We were unable to confirm previous findings of substantial importance of genetic polymorphisms in *GSTM1*, *GSTT1* and *NAT2* to chemical sensitivity, but the research data are an important reference open to further exploration. A possible weakness of our study design is the lack of assessment of the environmental exposure to chemicals metabolized by the enzymes we studied. In addition, the participants of this study were limited to men from the same work site, and the cases were not classified according to any confounding factors due to sample size restriction. In future studies, we hope to classify research subjects into multiple categories according to confounding factors in order to examine the relationship between genetic polymorphisms and chemical sensitivity.
